# Psychiatric Institutions and the Physical Environment: Combining Medical Architecture Methodologies and Architectural Morphology to Increase Our Understanding

**DOI:** 10.1155/2019/4076259

**Published:** 2019-01-06

**Authors:** Evangelia Chrysikou

**Affiliations:** The Bartlett Real Estate Institute, UCL, London WC1E 7HB, UK

## Abstract

The pluralism that characterized the development of psychiatric services around the world created a variety of policies, care models and building types, and fostered experimental approaches. Increased complexities of care, institutional remnants, stigma, and the limited diagnostic and interventional accuracy of psychiatric treatments resulted in institutional behaviors surviving, even in newly built facilities. This was raised by research on awarded psychiatric buildings. The locus of the research comprised two acute psychiatric wards in London. Each was evaluated using the SCP model, a tool specifically developed for the evaluation of mental health facilities, identifying the relation between policy, care regime, and patient-focused environment. Data were derived from plans, visits, and staff and patient interviews. Findings were juxtaposed to those of an earlier study using the same methodology. Also, a syntactic analysis was conducted, to identify the social logic of ward layouts. There were potential connections between regimes, spatial configuration, and the social fabric. Methodologies of architectural morphologies indicated areas that would attract people because of the layout rather than function. However, insights into medical architecture outlined institutional undercurrents and provided alternative interpretation to spatial analysis. Comprehending the social fabric of psychiatric facilities could challenge the current surveillance-led model, as psychosocial rehabilitation uses could be encouraged at points of higher integration.

## 1. Introduction

Mental health provision has historically been determined by perceived risks as those have been defined by each social context, rather than patients' needs [1]. It all started from the incarceration of the mentally ill people in a sequence of coercive institutions throughout western history as described by Fouqault [1]. Due to this coercive element, the power of the decision-making for the mentally ill belonged to the judges as they were responsible to keep society safe from potential dangerousness. This emphasis on dangerousness, on the protection of public and of judges being the people deciding for the fate of the mentally ill people, we could describe this model of care as jurisdictional. In the early 20th century, the gradual establishment of psychiatry created a shift. The judges were still powerful in responding to the inability of society to contain that risk, but gradually, mentally ill people left the mainly punitive “popper houses” and hid behind the visual impermeability of the newly established psychiatric hospitals. The position of the doctors over the judges became stronger towards the 1950s. The discovery of antipsychotic drugs challenged this custodial model [2]. The hope for cure enabled mentally ill people to be treated primarily as patients. This constituted a paradigm shift, establishing the medical model as the dominant model of care. Psychiatric patients were transferred to the psychiatric ward of the general hospital [3, 4]. However, soon drugs proved not to be a panacea, and the phenomenon of the revolving door phenomenon was observed [5]. This addressed the complexity of mental illness and the need for interdisciplinary therapeutic teams [6]. The model that followed, i.e., the psychosocial rehabilitation model, introduced various care options, located inside the community. Provision varied according to stages of illness, from crisis centers to vocational rehabilitation services and protected apartments, or personal needs providing from accommodation to socialization and peer support [7].

The variety of provision translated to variety of facility types, collaborating to each other and collectively responding to the spectrum of patients' needs. These networks were unique. Each responded to different financial, social, and organizational priorities operating locally and had rarely being copied elsewhere or at least not without adaptations.

This localized planning was encouraged by the principle of sectorisation [8, 9]. Established originally in France, it has not been named as such in other contexts. Yet, its essence of variety is a common attribute of mental health provision in the West [10, 11]. Under this principle, mental health authorities acquired considerable freedom to plan their own models of provision. This fostering of local variation, combined to the number of facility types, prevented the development of well-established, evidence-based design frameworks and typologies for psychiatric buildings [12, 13]. This paper explores the main concepts that led to the current psychiatric model in relation to corresponding psychiatric architecture practices. From this standpoint, it focuses on the area of developing fit for purpose methodological tools that could assist professionals involved in the planning, design, and evaluation of psychiatric buildings deliver solutions that are closer to care and patient well-being requirements. To achieve this aim, it examines evidence-based frameworks of planning and design of psychiatric facilities in relation to current therapeutic regimes and juxtaposes these specialized healthcare architecture frameworks to more generic methodological tools of architectural research. In other words, it juxtaposes frameworks developed in collaboration to patients and clinicians and relates to specific types of care in juxtaposition frameworks developed within the architectural establishment and is applicable to all types of built environment provision.

## 2. Community Mental Health Architecture in an Era of Radical Transformations

Defining the aims of healthcare architecture, Scher [14] urged the designer to support curing, healing, and caring via the physical environment. For psychiatric buildings, a similar approach of a supportive healthcare environment had already been introduced in 1960, by clinicians Baker and Sivadon in collaboration with an architect [15] and the World Health Organisation. They proposed a system of psychiatric care that included design guidance, with the psychiatric hospital becoming much smaller but still occupying a central role and being surrounded by an extensive network of facilities in the community. They fertilized design with psychosocial theories. The new architectural paradigm was characterized by therapeutic intentions and became known as “psychiatric architecture.” Therapeutic elements in space were sought through spatial situations, such as alternating between areas promoting a sense of security and insecurity, to trigger clients' emotions for therapeutic purposes [16]. Those theoretical concept designs feed the bipolar elements concept of Amiel [17]. He envisaged the hospital as one instrument among all the therapeutic tools the psychiatrist had and used the term “topotherapy” to emphasise the therapeutic properties of space [18]. However, these recommendations were very advanced for the crowded psychiatric wards of that period equipped with padded walls and immobile or very heavy furniture and were characterized by small openings [18].

The detailed guidance of Sivadon or Amiel was not expanded or materialized in the newly established community mental health facilities. Those were set up in diverse settings such as storefronts, office buildings, former private homes, or even self-contained luxurious clinics, providing in-patient and outpatient care, partial hospitalisation, emergencies, or consultation [19, 20]. In the UK, life in the community comprised the “hospital house” for stays of between six and twelve months, for people then to move to group homes, ordinary types of housing for five to six patients, with visiting staff [21, 22].

As community care progressed, the role of facilities had not been clearly defined especially on the transitory versus permanent characters [23]. There was also a growing disillusionment on the actual possibilities for independent life soon after patient discharge [24]. Similarly, the clinical environment of the general hospital, designed very much around invasive and complex diagnostic procedures, distinct flows, and extensive mechanical and electrical engineering requirements, was occasionally compared against leafier and roomier psychiatric campuses [25]. Soon psychiatrists and policymakers realised that the psychiatric institution resisted change, in line with Zucker's theory of institutional resistance to change [26]. Despite the fact that patients were moved from old asylums general hospital wards, institutional practices could still survive, even in the new premises. Therefore, modernization should also include the social environment of the facilities [27]. Even the ability of psychiatrists to diagnose partially discredited clinical settings, favoring the voices of antipsychiatry movement [28]. However, soon there were indications that facilities in the community could cultivate institutional behaviors, making clear that psychiatric facilities incorporated a significant level of sociospatial complexity [25, 29].

Here, we need to mention that the efforts of defining psychiatric space between the two poles of hospital and home constituted a discussion mostly among psychiatrists and healthcare professionals. The models of medical architecture could offer little help to address the following issues: this was because a) they focused on the increasing clinical and technological complexity of the general hospital, where most psychiatric wards sat, b) the sift of mental health provision towards the community constituted the hospital ward typologies irrelevant.

To bridge the gap, “normalisation theory” was introduced to mental healthcare. Normalisation was derived from the field of learning disability, where it was and remains an established theory [30]. In short, it opposed institutionalization by promoting everyday-life practices to people with disabilities and lifestyles similar to that of the normative population [31, 32]. Translation to design principles normalization implied “homelike” environments. Nevertheless, it lacked a definition of what is homelike. Goodman [33] supported normalization speculating that the building served clinical purposes by providing a normal environment, with respect to clinical needs. He criticized Amiel and suggested that user involvement, cost-effectiveness, and integration to the locality should be above architectural gestures. Normality to him was a combination of brickwork, normal furniture, and carpeting, even if security had to be compromised as a result.

Towards the last quarters of the last century, three options of care coexisted: (a) ordinary housing for all, (b) care in the community but in cooperation with a hospital, and (c) modernised hospital care with its own community network [34]. So, when in the 90s medical architecture shifted from functionalism towards patient-focused environments, psychiatric theories could add to the dialogue by addressing the decentralisation of provision and our limited and fragmented understanding of therapeutic space.

This diversity and limited interdisciplinary relations between architectural practice and health sciences created an experimental, intuitive as opposed to evidence-based, approach regarding the design of psychiatric facilities. This has been rapidly changing in the last decade [35], the case for the most psychiatric building stock.

Additionally, one should consider the following:

- The increased complexities of psychiatric care, including limited diagnostic and interventional accuracy of mental health conditions [36].
- Institutional residues in community mental health facilities. Bricks and mortar alone could not fully address social reintegration [37, 38].
- The stigma [1]. Mental illness has been described as the most stigmatized illness, even by healthcare professionals and within the healthcare system [39, 40].

This combination resulted in an array of institutional behaviors and practices still surviving in purpose-built facilities or new, community-based buildings. Lack of knowledge of how space operates might lead to build environments that generate social problems [41]. The more vulnerable the user group is, the stronger the need becomes to develop and use knowledge-based design tools [42]. This is of high importance in mental health settings since they present social problems such as violence and substance abuse [43].

The gap of knowledge on psychiatric space was accentuated when interdisciplinary research combining methodologies deriving from medical sociology and architecture found that even awarded psychiatric facilities might present strong institutional characteristics regarding building features and in terms of users' perspective [44]. The research took place in the early 2000s, when there was a significant gap in research on psychiatric environments. These case study buildings were designed with the best of intensions and awarded as state of the art, innovative and challenging boundaries of design for mental health. However, four main reasons led them to acquire scores closer to the institutional end in an institutional vs domestic scale when compared to other facilities and at the same time gathered a fair number of complaints from patients and staff. First, there was scarce evidence regarding the design of psychiatric facilities, with no strong causal links between design and clinical outcomes [45]. Also, deintitutionalisation was relatively a new practice. Therefore, there was a lag between this concept and the development of an architectural theory that could cater specifically for the spatial requirements of these new community mental health facilities. The model that was covering that gap was normalization, and it was developed for a different healthcare discipline. Yet, that direct implementation of normalization to mental health compromised the needs of mentally ill people in the most acute spectrum of care provision [46]. Coming from healthcare architecture practice, Mungo Smith pointed the right balance between privacy and surveillance as a major dilemma for the design of psychiatric facilities at the time [47]. Third, there has been a lack of evidence-based culture in architectural competitions in general, even in those for healthcare [48]. Finally, there was absence of service-user involvement and vertical advocacy of staff in the decision-making at planning stages. This resulted in disparity between the psychiatric principle of psychosocial rehabilitation, architecture, and user expectations or between the numbers of human resources in these facilities. It was also at odds with former practices of bottom up initiatives established as early in the mid of the previous century. These favored user involvement in the planning or running of the facilities or even were entirely user-led organisations and were drivers of the whole rehabilitation movement [49]. Also, user involvement at the design stage has been associated with wards with higher patients and staff satisfactions [11, 50]. Patient involvement and collaborative practices also help in creating a sense of community within psychiatric institutions, an upcoming approach within violence management and with the potential to replace coercive measures [51].

These generated the question on the relation of the building layout to psychosocial performance, together with requirements related to pathology and potential therapeutic aspects. Translated to research aims, this research set the following objectives:

- To explore the spatial dynamics of psychiatric institutions and how those influence (a) the personal and (b) the social milieu of psychiatric space
- To identify environmental requirements of mentally ill people according to needs, therapeutic regimes, and principles deriving from deinstitutionalization

## 3. Methodology

### 3.1. Towards an Integrated Model of Mental Health Facilities Design

**Figure 1:**
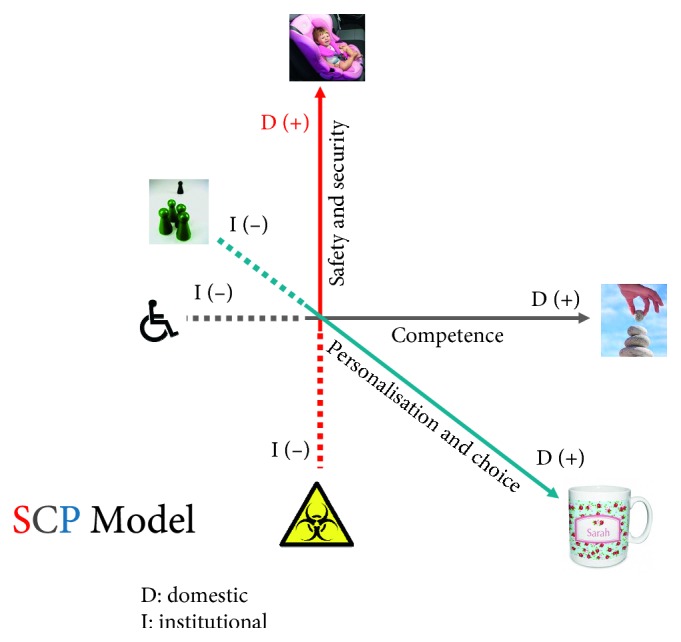
The SCP model as a 3D space where psychiatric facilities can be placed according to their individual characteristics in domestic (+) vs institutional (−) scale.

**Figure 2:**
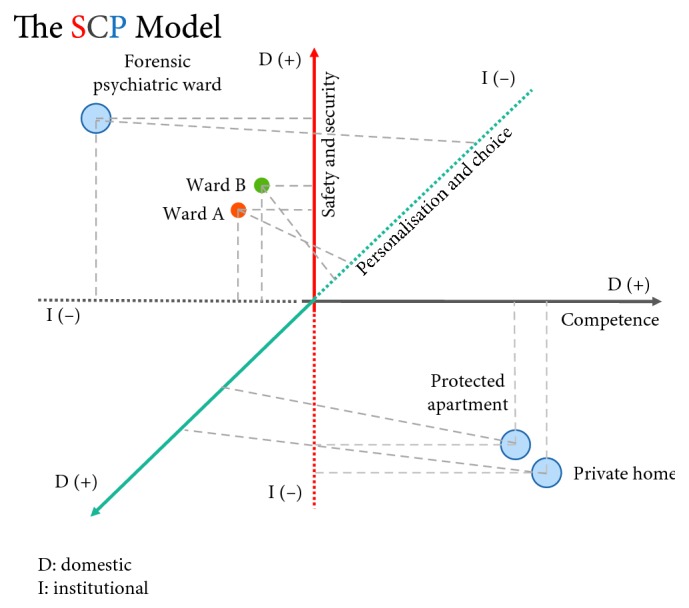
The case studies projected on the SCP model in relation to the spectrum of the mental healthcare building stock.

For the first part of the first objective of spatial dynamics of psychiatric facilities in relation to the personal milieu of the patients, the research was built on the previous line of work of the PI. It involved a model specifically designed for the planning, design, and evaluation of psychiatric facilities based on medical architecture principles [46]. This was the SCP model, named after the acronyms of three variables: safety and security, competence, and finally personalization and choice. It was a three-dimensional model, and each of these variables comprises one dimension of a cubic problem space occupied by three axes (*x*, *y*, and *z*) (Figure 1), where safety and security implies an opposite pole, where the building is unsafe and insecure, where competence implies a situation where dependency is fostered in patients, and where personalization and choice also implies a situation where no personalization and choice is allowed. Each building could theoretically occupy a unique position in the three-dimensional problem space of the model, which is therefore both more sensitive and more specific than the polar opposition between domesticity and institutionalization, previously described [11] (Figure 2). The model could depict the quality of environment and its consequences to patients' life.

**Figure 3:**
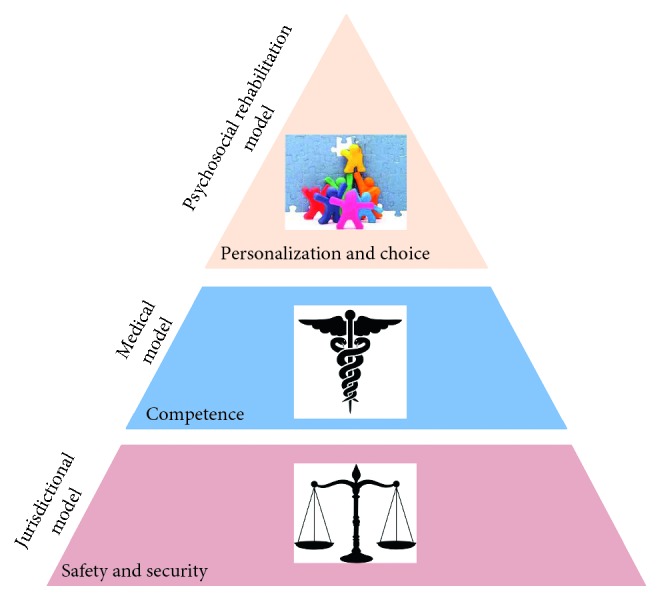
The SCP model and the pyramid of needs: each tier represents a parameter of the model (named by the acronyms of the three parameters) and corresponds to a model of mental health provision.

Additionally, the model incorporated the key issues behind mental illness expressed by the dominant models of care as they evolved over the years. These three main concepts, the jurisdictional, the medical, and the psychosocial rehabilitation model, were described in the introduction of this paper, and each corresponds to one of the parameters (Figure 3).

Even though the shift has changed over the years from the custodial to the psychosocial rehabilitation models, elements of each still exist in each psychiatric structure. The relationship of each facility or each care program to each of the three defines where each facility sits between the three: for example, a forensic facility might appear closer to the jurisdictional model, and a service-user operated café is closer to the concept of psychosocial rehabilitation. Yet, each displays elements that belong to the other two. For example, a forensic facility might have an occupational or music therapy room, or the rehabilitation café might be supervised by a clinician.

From the above, it is clear that all topics on mental health environments could be classified according to these parameters. These derived from the basic needs related to mental health priorities as they relate to the main objectives of care:

- Harm and self-harm prevention (essential for existence and therefore forming the basis of the pyramid) and corresponds to safety and security
- Medical and nursing provision, to restore competence compromised by mental illness
- Social reintegration, promoting the personalization and choice that are lost in institutional environments and correspond to well-being

The SCP model could help define enabling environments which staff consider as best practice and patients perceive as suitable for their environments of care. So far, the SCP model has been already applied in facilities in the UK and in France [11]. Data used in the previous study could add longitudinal evidence of changes that happened, bearing in mind though the limitations as the facilities in both studies are of the same time but not the same buildings and neither belong to the same health authorities. The model concentrates on patients in relation to care models and needs.

Therefore, it has limitations regarding the sociospatial dynamics inside the wards.

To address these sociospatial dynamics, a second method would be needed. One of the most widespread architectural theories associated with sociospatial dynamics is the theory of space syntax [41, 52]. This theory of architectural morphology has developed tools that could look in more depth on the opportunities for social encounters that buildings generate. In this case, it would investigate the sociospatial relations and dynamics of space. This methodology has been widely used in urban and normative architecture settings but was seldom used in healthcare settings and in particular psychiatric. The question would be if we could set more light to the sociospatial angle of the institutions and if we could identify any generators of institutional environments. Thus, this project for the first time brought together the two frameworks, i.e., the SCP model and Space Syntax [41], the former designed especially for mental healthcare and the latter for all spatial scale.

Space Syntax is a sociospatial theory with a strong algorithmic toolset, involving all scales of planning and design and all typologies looking at their spatial structure. According to the theoretical framework of Space Syntax, the relation of space and society is interconnected. Hillier [52] suggests that, for research buildings, we need to find their cultural and social patterns, through their nondiscursive contents. This is because human understanding of spatial configurations happens intuitively and we do not have the vocabulary or discursive mechanisms to express and therefore research it. Netto [53] describes among the strengths of Space Syntax its understanding on social reproduction, copresence, and the embodiment of practice, its relational concept of space, and the reaffirmation of space as a living dimension. The main question of Space Syntax could be described as the influence of urban configuration on society [54]. As Westin [55] sums up, Space Syntax does not support that built environment determines people's interaction with each other but rather that the environment through spatial configuration creates/disables opportunities for social interaction. This is something that researchers should bear in mind regarding healthcare facilities as different people, especially when they have less ability to cope because of illness or disease, may experience ward design in conflicting ways [45].

The emphasis on layout irrespectively to other qualities of placemaking that the SCP model addresses is an important reason of including Space Syntax in this project: data occurring from spatial analysis would be ‘unpolluted' from preconceptions of medical architecture.

Space Syntax being a generic sociospatial theory has been mostly employed in urban studies. Nevertheless, it starts to be applied in healthcare [56–60]. However, only in the work of Hanson with Zako, the research has been juxtaposed to concepts of medical architecture such as patient-focused environments. In most research in healthcare settings that employed Space Syntax, healthcare facilities were researched as buildings rather than systemic parts of health services that constitute a major principle of healthcare engineering research. This focuses on spatial configuration only without considering the neurodiversity or visual competence of the sample and might be a strong limitation of these projects, limiting the validity of the results. For example, Space Syntax might present serious challenges when peripheral vision is limited or even more if people present visual impairment, or in general the physiological and perception limitations of the participants, including results of sleep deprivation, stress, or medication. This has been also the case not only in medical settings but in all settings when viewed from the perspective of disability in general, as they do not address elements such as walkability [61, 62].

By combining these two methodologies, the research set to investigate both patients' relation to the therapeutic regime and social relations to the spatial configuration. The SCP model constituted the basis of the evaluation, being more high-level in the aspects covered even though it is less generic when it comes to population and the building types, since it has been specifically designed for mental health. Space Syntax, on the contrary, has broader applicability in the build environment but is more focused on layout issues and does not cover issues such as fixtures and fittings, technologies, availability, and types of human resources or aesthetics. Thus, it provided tools on observability, wayfinding, and social solidarity. The findings from that methodology enriched areas that come under the SCP parameters. The morphological analysis is used for the first time in psychiatric wards, yet a meta-analysis of earlier research using the SCP model is possible. This could extend the sample and provide a clear understanding to the methodology, especially if performed to the awarded case studies of the previous UK sample of the SCP model research. However, this is an area for further research and remains beyond the scope of this paper. Findings of agreement between the two frameworks enable the formulation of an integrated model for mental health design, and findings of nonagreement allow these theories to evolve by addressing the limitations that the research pointed out.

Regarding the objective on the built environment in relation to psychiatric space, the research sets to establish a valid framework of designing for mental health. To achieve that, this user-inclusive research involves academics and architects, health authorities, staff, and patients. The locus involved two secure acute facilities chosen according to preset criteria and permission granting, in line with the growing policy of community care. For the secure, acute parameter, the more severe the symptomatology is and the closer to the acute episode id, the more important the therapeutic environment is, and the more persistent the institutional regime might be.

### 3.2. Tools

The main tools used for the research comprised the following:

- For the SCP model: a staff questionnaire, a patient questionnaire, a building trait checklist
- For the spatial analysis: Depthmap and JASS tools on blueprints

### 3.3. Interviews

For evaluating patients' needs and the compliance to care regime, patients and staff were interviewed by the researcher using semistructured interview questionnaires of 30 and 23 main sets of questions, respectively. These questionnaires were designed, piloted, and used by the same researcher for the previous research on the UK and France community mental health facilities, in the early 2000s. It comprised three subsets of questions, each referring to one of the three parameters (security vs autonomy, competence vs dependency, and restrictions vs opportunities for personalization and choice). The topics were derived from literature on psychiatric rehabilitation and policies related to psychiatric inpatient environments and were then translated to spatial implications that were then checked with patients and staff.

Architectural auditing: data on the physical environment and sense of place of the wards derived from a systematic architecture account for spatial organization, therapeutic regime, salutogenic qualities, i.e., the building qualities that enhance health [63–65] such as day lighting, art, natural views, and access to nature based on visits, photographical auditing, and plans. Regarding the plans, architectural blueprints were compared on their analogies of areas per use and user group. The architectural auditing was conducted by the researcher.

Checklist: third, a detailed architectural checklist, part of the SCP methodology, was used to identify the “normal” as opposed to the institutional traits of the buildings. The checklist dissecting each building to 212 traits identified institutional physical characteristics in a comparative scale to the local norm as defined by the neighboring or local residential buildings in parameters related to the exterior, the layout, and the design of the interior. It was the same checklist that was developed for the UK-France study and had been adapted by a similar checklist of Robinson et al. [66] researching residential environments for learning disabilities. With the use of the checklist, each building could acquire a unique score in terms of institutional versus domestic architectural traits, either or as a part and could be directly compared to another building using the same scoring system. The checklists were completed by the researcher at the end of each visit.

### 3.4. Depthmap and JASS Analysis

Finally, regarding architectural morphology, each facility has been examined using Space Syntax, a theory focused on the research and analysis of buildings as patterns of space inhabited by individuals, communities, and organizations (Table 1). Space Syntax tools used for this research involved social solidarities, social relations diagrams, and integration values. The morphological analysis was performed for the interior of the facilities, using Depthmap software [67] on blueprints of the current state of the wards. The tools used comprised convex analysis, axial analysis, visibility graph analysis [68], and JASS Software for justified graphs.

### 3.5. Procedure

The selection of the case studies was done by the two participating Trusts. The inclusion criteria comprised each facility serving the most acute spectrum of care, being for inpatients and not being part of the forensic mental health system. As the research had a limited time span of two years including ethics approvals, timing was crucial.

For interviews, the ward manager approached staff and patients and distributed information about the project, after prior discussion with the ward psychiatrist on which patients were well enough to participate. The researcher then interviewed each staff and patient willing to participate. Each participant was given the ethics material to read, and the interviews took place after the consent form was signed by each participant. Interviews took place in a room that could offer auditory, but in the case of patients having visual permeability, in the corridor. For patients, it was the quiet room for Ward A and either the dining room or a consultation room for Ward B, depending on availability. For staff, there were some additional locations: the staff meeting room in Ward A and the nursing stations in both wards. Each interview lasts approximately from half to one hour, depending on participants' willingness to talk. The interviews were conducted by the PI of the project. The transcripts of the interviews were done by the PI.

To avoid the researchers' bias in the ward selection process and the influence of building aesthetics, sites were chosen by the two participating Trusts.

The researcher kept a detailed photographic record of the physical environment of the wards and was escorted by a member of staff in all common ward and staff areas and a selective number of bedrooms, mostly those not occupied at the time.

### 3.6. Participants

Ward A accommodated 22 patients and Ward B 15. For patients involved, staff suggested a potential pool of patients who were well enough to participate. Then, it was up to these patients to decide if they wanted to take part. Staff recruitment was related to staff availability. In the total of 11 patients, 4 from Ward A and 7 from Ward B were well enough for staff to allow them to take part and at the same time people willing to participate were 10 members of staff, five from each ward. Due to the facts that the potential sample was rather small, the results to protect participants' identity and the ethics approval individual characteristics, such as name, age, gender, and exact pathology, were not asked. All patients belonged to the acute spectrum of psychiatric illness requiring hospitalization, and Ward A was for a single gender. The stage of the illness—closer to the acute episode vs closer to independent life—was the main factor that could affect preferences compared to age or gender in the previous study [11] with patients being less able to cope from non-fit-for purpose design. Therefore, this study focused on this patient group.

### 3.7. Inclusion Criteria

The inclusion criteria for service users were that they were adults, living in the wards at the time of the visits, able to give informed consent and willing to participate. All staff who would volunteer were eligible for the study, as long as they were working in the facilities at the time of the visits.

### 3.8. Sites

The locus of the research comprised two acute psychiatric wards in London, belonging to different Mental Health Trusts, all part of the public healthcare sector. For Space Syntax research, samples in hospital research comprise most of the times one to three settings. In this case, the original sample was three London wards but delays from one of the facilities plus additional requirements for the Ethics approvals of that one caused delays that lead to the elimination of the third case study. One of the rest case studies presented substantial delays but which were not so severe as to eliminate it from the sample, due to a fire incident which led to the need to evacuate and renovate the ward. The field work took place a month and a half after the residents were back in the ward.

**Figure 4:**
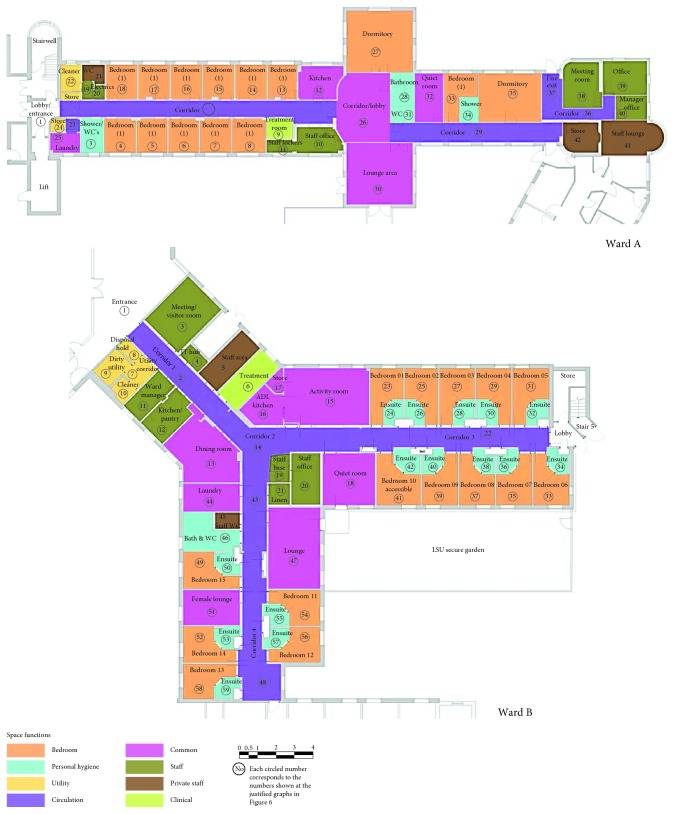
Floor plans of Wards A and B. The architectural drawings are color-coded according to functions.

Despite the UK having officially closed its psychiatric hospitals, following a series of legislation since the 60s, both wards were situated inside larger psychiatric complexes. Their facades bear strong visual references to nonresidential architecture even though both are part of care in the community. Case study A (Figure 4) was part of a former fever hospital campus, yet it has been converted to a mental health site for four decades. The entire campus has strong institutional characteristics and is surrounded by a tall brick wall, even though the campus entrance is open. The condition is poor. Pending plans to redevelop the entire campus span are present for more than two decades, and investment has been halted for that reason. On the contrary, case study B (Figure 4) sits in a recently remodeled Mental Health Center. Both wards sit on the ground floor, although in both complexes, other wards sit on floors and are deprived from direct access outdoors. Thus, residents receive remodeled mental health center and a recent fire incident in the ward meant that it had been renovated weeks before the field work was conducted.

### 3.9. Ethics

The project was carried out following all ethical procedures and permission required by the Research Ethics Committee (REC) of the National Health Service (NHS) with REC Reference Number 15/LO/1297. Informed consent was obtained from both patients and staff after they had been informed orally and in written by a member of staff.

Participants retained their anonymity throughout the project.

## 4. Results

### 4.1. Results from the Checklist

Both wards present a strong institutional character: an average of 60.85% and 54.72% according to the Institutional vs Domestic Checklist for Wards A and B, respectively (Table 2). In terms of layout (building features), the wards have identical number of institutional features. Regarding the surrounding area and exterior (context and site), Ward A presents more institutional features than Ward B, and this is similar when it comes to the interior design of each room in the building (space and room features according to the Robinson classification [66]).

### 4.2. Results of Auditing

Regarding layouts, the two wards presented similarities yet there are key differences too (Table 3).

Differences constituted the placement of the offices, the self-sufficiency of the wards, and the provision regarding sharing accommodation and toilet facilities. Also, they differed regarding gender segregation arrangements and challenges imposed by not only smoking policies but also the challenges that the policy would create to the internal configuration of the wards. Regarding wear and tear, Ward B was recently renovated.

Overall, Ward A presented wear and tear and demonstrated institutional traits such as dormitories and shared toilets, considered obsolete. On the contrary, Ward B was maintained in an excellent condition. Still, the number of its institutional traits was comparable to that of Ward A. This, however, was the result of an extensive use of antiligature fixtures and fittings combined with compromise of salutogenic elements, such as removing the sheltered outdoors area, as it was perceived dangerous.

**Figure 5:**
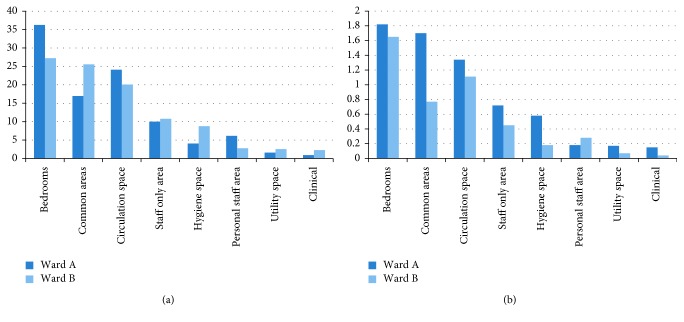
Comparative chart of (a) ratios of areas in the two wards and (b) ratios of areas in the two wards per patient.

Ward A was more spacious per patient than Ward B (Figure 5). The only part that Ward B appeared more spacious was common areas but not per capita. This involves only the area within the ward and not neighboring areas that were used on daily basis such as the dining room but was not part of the ward. Additionally, Ward A had a larger internal garden where patients could visit all the time.

### 4.3. Interviews

Interviews provided important qualitative data. For example, passive behavior is mostly met in Ward A, and activities are mostly common in the case study for Ward B. Willingness to participate in interviews was also higher in Ward B. Recruitment in Ward A was challenging, even though potential numbers for patient recruitment were higher. Dormitories as opposed to single rooms seemed to increase passive behavior combined to the limited availability of staff. So, several patients that were lying passive in their beds or sleeping—a typical characteristic of an asylum rather than a community mental health ward in care in the community—refused to engage in interviews. In one occasion, staff urged a patient to wake up and participate but the person was not in a position to participate (complete lack of concentration and inability to engage), so the researcher did not proceed with the interview. In general, both staff and patients in Ward B reported positive and frequent interaction between themselves. It is not possible to discuss all findings from the interviews in one paper. We will briefly refer to two findings that are relevant to the discussion below, as they relate to the space syntax analysis. One is the control from the nursing station, a staff only question. The second is a shared question and relates to the smoking policy.

Staff interviewed on the nursing station, and its effectiveness in providing adequate control was 7/10 satisfied. There were 3 staff, two in Ward B and one in Ward A who were dissatisfied with the control available from the nursing station. The latter pointed that the nursing station felt suffocating.

One of the very interesting differences was the smoking policy. Ward B did not allow smoking in the campus, so patients had to get escorted to leave to smoke, one patient at a time. The majority of staff and patients in both wards agreed with smoking in the premises, although the majority of staff supporting smoking inside the courtyard of the ward was smaller in Ward B. There 3/5 supported smoking in the courtyard, and one of whom claimed that the previous fire was related to the smoking ban as lighters became then a rare “commodity,” and patients hid them. In Ward A, all staff supported smoking in the courtyard, and one was concerned that the policy would have to change soon to nonsmoking. He acknowledged that smoking is not benefiting patients' health, but at the same time, hospitalization is a tough period to stop smoking, a position that has also been reported in the literature [69].

### 4.4. Space Syntax Analysis

**Figure 6:**
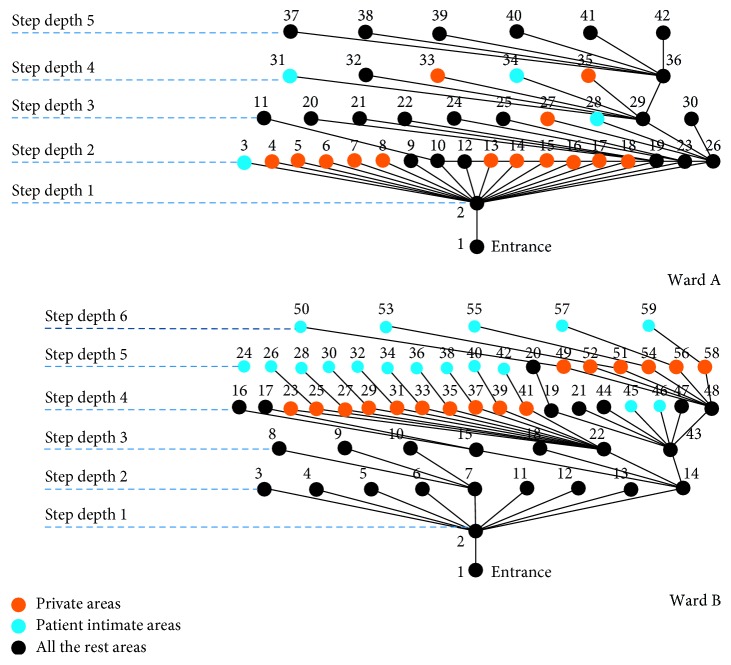
Justified graphs of wards showing depths of private/intimate areas.

**Figure 7:**
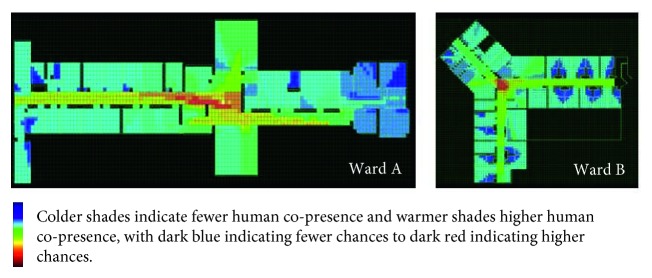
Integration of Wards A and B. The colors are computer generated from the shape of the plans and indicate chances of human copresence (from dark blue indicating fewer chances to dark red indicating higher chances).

The justified graphs present the spatial configuration of the wards. The two justified graphs (Figure 6) present similarities in their overall shape and relatively similar amount of depth. Yet, there is a considerable difference between the two wards on the placement of the private and intimate areas. In Ward A, those appear in the same depth with the rest of the ward areas. This is a sign of a nondomestic (institutional) architecture. In Ward B, we see that those are placed in deepest parts of the building, i.e., hidden from the entrance and from public spaces. This gives better control of the “inhabitant” (patient) in relation to the “visitor” (staff), according to Hillier and Hanson [41]. Therefore, the typologies appear similar. However, the placement of private and intimate areas in the justified graph of Ward B presents higher resemblance to domestic buildings and a greater sense of normality. The difference of the depth of the intimate and private areas in the two buildings is very visible in Figure 7, where the deepest parts (dark blue) in Ward B are in the toilet section, followed by bedrooms (lighter blue), but for Ward A, the deepest areas are mostly in staff offices. In both wards, the warmest area (dark red) is in the corridor, just outside the nursing station.

From the plans, it shows that the most integrated space is the area outside the nursing station (red)

## 5. Discussion

Overall, the research produced a significant volume of data, deriving from the checklist, the architectural auditing, the spatial morphology analysis, and the interviews. These generated a comprehensive series of findings regarding the architectural features of the buildings, the therapeutic regimes, the layouts, the relationship to care models, and to users' preferences, plus their relationship to the data of the early 2000s UK research [11, 46, 50] that used the SCP model and generated a comparable amount of data that could not be presented in a single paper.

The wards presented similarities when using the checklist and Space Syntax methodologies contrary to visual inspection of the plans (Ward A layout appears linear, as opposed to the axial shape of B). Ward B presented an added level of depth and also provided a more domestic one as opposed to institutional layout in relation to private and intimate areas. The relatively central positions of intimate areas of Ward A, with easy access to the core of the ward (in this case, the ward corridor) but without direct access to the private areas, which is more normal and protects patient's privacy, bear references to the medical model and the old general hospital wards. In Ward B, the most segregated areas allowed the highest privacy. As they were also en-suite, they reflect the psychosocial model but also trends in patient-focused ward design for general hospitals.

Nevertheless, having a closer look at the complexity of those data, we could certainly refer that the number of institutional features alone could not convey the character of each facility.

The wards are at the institutional end or very close to it when compared to the former UK sample, i.e., of the case studies that had been investigated using the same checklist 15 years ago (Table 4) [46]. This is even the case in the renovated ward.

The strong emphasis on suicide prevention through design is one of the clear differences between the original research conducted using the SCP method in the UK in early 2000s, together with a clearer gender segregation. The move towards increased antiligature and gender segregation agrees with the National Service Framework (NSF) for Mental Health [70]. In fact, these are the two points that NSF mentions as important for the environment of the wards and opposed to the gender integration and homelike fixtures and fittings than the normalization model advocated and was the dominant framework during the earlier study [46]. This shift of direction highlights the dilemmas around the priorities of psychiatric services as expressed by the dominant care models or the SCP parameters that relate to them. In this case, the dilemma between the safety-security (jurisdictional model) axis and the personalization and choice occurred from the psychosocial rehabilitation model of care: the antiligature and segregation vs more normal environments both in gender interaction and ordinary fixtures and fittings (for example, ordinary taps with mixers that provide better shower experience but bear weight). Studies support that homelike features together with opportunities for privacy increase social interaction and support well-being [45], yet, at the recent years, the use of increased antiligature means increased suicide rates in inpatient psychiatric declined. Yet, research in Finland associates similar reduction to other reasons, including more outpatient treatments and more effective treatments [71] and earlier UK studies associate the decline with better aftercare and improved service provision [72]. Moreover, research in German wards suggested that locked wards when compared to open facilities do not seem justified to prevent suicide and absconding, although due to differences in the service provision constitute the German inpatient sample less severely ill compared to the UK one [73]. Ward A bore considerable references with institutions yet scored the same in terms of layout and notsignificantly worse in terms of exterior and interior design in terms of institutional features compared to Ward B. The latter incorporated several elements of the state of the art technologies in antiligature psychiatric design, but this was to minimise ligature rather than increasing the social reintegration of its residents. Thus, in the same geographical area, i.e., London, we could identify two distinct models of care provision: one prenormalization providing low stimulation, limited privacy, and sociofugal design of sitting arrangements [74, 75] and one postnormalization featuring specialized psychiatric design, especially in terms of materiality. The latter could be described as a reintroduction of the psychiatric ward of the general hospital in the community: emphasis on infection control, antiligature, central nursing station, provision for various degrees of gender segregation, and general hospital policies such as the nonsmoking policies in all hospital outdoor areas, etc. This agrees with the conclusion of Killaspy [76] after reviewing psychiatric literature on deinstitutionalization that community services could not completely replace hospital care resulting in increasing reinstitutionalization. The need for specialized settings, as opposed to mainstream spaces in the community, have also been advocated to provide psychosocially encouraging spaces, even if these initially appear to provide less opportunity for activity or social interaction than mainstream spaces in the community, which however could be intimidating to patients, and therefore, this extra opportunity might remain unexploited [77].

The qualitative characteristics are mostly depicted by the analysis according to the three parameters of the SCP model (where we can see, for example, where the ward site is compared to antiligature, medical, or the rehabilitation models), the architectural morphology analysis, and in particular, the analysis of user hierarchies (for example, the integration value of the staff areas compared to the integration values of the rest areas) and policies such as gender segregation, smoking policy, or access to the existing outdoor areas.

Higher percentages of private areas did not prevent that, on the contrary (Figure 5). More gradual transitions of private and intimate areas, i.e., deeper in the building compared to public areas, and promoting dignity in Ward B could be also a contributor to an increased sense of well-being compared to Ward A samples (Figure 5). Having said that, Ward B was recently renovated, a factor that might have influenced positively the reactions of staff.

From those differences, it is worth looking at the smoking policy. This is a delicate point as smoking is harmful for patients' health. Yet, being forced to stop at a time one had to deal with a crisis and involuntary admission could be adding to stress. This resulted in long queues for escorted leave to smoke, unrest, and incidents of violence outside the nursing station and had been the reason behind the fire according to the ward manager. The participants, staff, and patients were in favor of patients smoking in the courtyard as opposed to leaving the premises. Smoking policy might be related to cultural and local trends, although a survey among staff in New York indicated that staff tend to change the position regarding smoking areas lately, towards smoke-free environments [78].

Therefore,

- One less institutional facility (Ward B) in terms of building features might demonstrate a strong institutional behavior, such as institutional queuing outside the nursing station like former asylum practice of “cigarette distribution” by a specific policy.
- Social unrest can be created by policies (such as the nonsmoking policy in Ward B) even if buildings have provided solutions (such as an enclosed courtyard).
- Policy and buildings in mental health facilities are interrelated. Yet, policies might change at any time during the building life circle.
- Policy might affect the spatial use of mental health facilities considerably.

**Figure 8:**
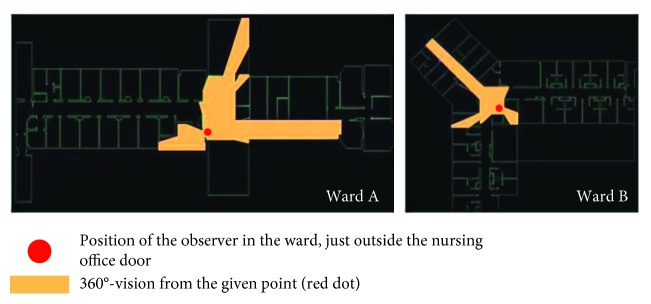
Visibility from the nursing station at Wards A and B. The dot on the two graphs represents the position of the observer in the ward. The raster shows the 360° vision from that given point.

The integration of the nursing station could generate food for thought regarding the application of Space Syntax in institutions. What Hiller and Hanson [41] would describe as social logic of space might be severely compromised by top down imposed rules, restricting movement, such as access to offices or curfews, as well as imposing non-natural movements, such as the escorted passing through complex indoor and outdoor routes in the campus to reach an open area that was not owned by the National Health Service (NHS) to smoke compared to the more intuitive option of accessing the ward courtyard. On the contrary, Space Syntax detected as spaces with the potential to generate social interaction in those locations where patients tended to demonstrate strong institutional behaviours such as queuing and antisocial outbreaks, which contradicts the Space Syntax findings for these spaces. For instance, in both wards, the most integrated spaces appear to be the spaces outside the nursing station (Figure 7). They are also areas of visibility (Figure 8). Similar to total institutions, patients gathered outside both nursing stations putting themselves in the surveillance “radar.” Visibility from those points might have been requirements of the architectural briefs. Yet, most staff were not present there. Patients did not gather outside the staff office of Ward B, which was at a segregated part, neither outside the entrance connecting the ward corridor to the staff only part in Ward A, which was also segregated. Patients gathered at the most integrated point. It remains uncertain whether that was a demonstration of an institutional behavior or a human need of meeting people at the point that spatially provided the highest chance of social encounters. The corelation between the two areas of high integration and antisocial behaviors contradicts the Space Syntax theory. Yet, if we combine the high scoring in institutional features, then perhaps we could suggest that it is because these wards have strong institutional elements according to Goffman's theory on total institutions [79] that they would operate against norms. In this case, these buildings might fit into what Hanson and Hillier would describe as “inverse” types, a terminology they use for institutions [41]. This could be the case in Ward A, as private and intimate areas for inhabitants (patients) are not in the deepest part of the axial diagram. However, in Ward B, the axial graph does not justify this, as private and intimate areas are in the deepest levels of a building, very similar to normative accommodation.

The above becomes more complex when we consider the visibility from the nursing stations. One of the key aims of a nursing station is surveillance (Figure 8). However, visibility from the nursing station glazing to the corridors in both cases had been partially blocked by staff, and in both cases, staff had their backs to the corridors. Thus, staff could not obtain visual control. This questions the centrality of the placement of the nursing station, one of the key spatial features of institutional design dating as back in the history of mental health design as the design of panopticon, in terms of a briefing priority.

## 6. Conclusions

Dangerousness and perception of risk for harm or self-harm still dominate the design of mental health facilities in the UK. This is the case despite the optimism that surrounded psychiatric rehabilitation movements. The paper presented findings highlighting potential connections between policies, care-regimes, spatial configuration, and the social fabric in psychiatric institutions. The research combined the SCP model, a tool specifically developed for the evaluation of mental health facilities to Space Syntax, a generic methodology that identifies the potential for socialization that spaces generate. These methodologies of architectural morphologies indicated areas that would attract people because of the layout rather than the function (Figure 6). However, in institutional contexts, this was influenced by the social fabric of the institution, i.e., generating institutional behaviors, instead of sociospatial interaction.

This indicates that generic methodologies such as Space Syntax used without involving tools deriving either from medical humanities such as medical architecture and medical sociology or from a more clinical or healthcare management perspective could not provide results that could be used with confidence, as the healthcare context is a much more controlled and multiparametric environment than normative urban or architectural planning. In the case of the psychiatric facilities, this research demonstrated that Space Syntax produced inverse results of what was actually happening by indicating as areas of the social value the areas where antisocial behaviors used to take place. This could be interpreted as inadequacy of Space Syntax to cover complex multiparametric settings—especially as Space Syntax takes a minimal number of parameters into account—but also could mean that psychiatric facilities are still what Goffman named “total institutions” [79]. At the same time, it highlights considerable limitations of Space Syntax algorithms when it comes to healthcare facilities [80–82] or when the research population is not normative [83, 84].

The insights into medical architecture and healthcare facilities planning can outline institutional undercurrents and help make better sense of the spatial analysis, which on its own can been misleading. This is in agreement with the growing trend of employing comparative methodologies to conduct research in healthcare buildings [57].

The most important finding is better understanding on the dynamics of the psychiatric institutions in general, even when care in the community policy suggests that institutions have been abolished by downsizing. This project indicates that this has not been achieved yet. As a result, it challenges the way psychiatric buildings are planned and designed from the current surveillance-led model to integrated design for patient well-being. From a clinical perspective, this would mean that psychosocial rehabilitation uses could be encouraged at points of higher integration. This would be beneficial for all user groups and mostly the actual recipients of care.

Regarding limitations, the interconnection between design and social logic results in difficulty to establish cause and effect. Future research involving more case studies that vary considerably in spatial configurations, institutional levels, and regimes could gradually provide better understanding on the key determinants. To move to the next level, we need a larger sample of wards with significantly different layouts, and spatial configuration could indicate more accurately a relation between the most integrated areas and higher chances of patient copresence. If such a relationship could be established, then spatial planning should consider placing activity or the social areas in zones of potential high spatial integration. It would also be important to investigate a potential association between behaviors and institutional features. These could provide new insights into psychosocially supportive ways of designing.

Due to the complexity of this methodology, especially the significant involvement of very vulnerable patients, adopting a larger scale approach might be challenging. UK wards also tend to be variant and small with a limited number of patients and an even smaller number that would be well enough and willing to participate and limited staff. The fact that they differ in policies increase the complexity further. However, a larger scale study using this methodology would provide a considerable amount of data that could give a very comprehensive understanding of several aspects of the planning and the design of psychiatric facilities and especially those of the most acute spectrum.

The experience of people living or working in psychiatric facilities and their interpersonal relations, health, and well-being are influenced by their environment. The research provides the ground for an integrated design framework for evidence-based mental health architecture to serve as a design and evaluation tool, immediately accessible to architects, planners, and stakeholders. It contributes to the growing sector of evidence base design, informing body of knowledge that aims at improving healthcare conditions for patients and the efficiency of facilities through design. Incorporating the full spectrum of patients' needs (physical, caring, and well-being) and recognizing spaces as cells that allow mechanisms to operate and influence behavior towards social integration, unlike institutions [85], could inspire more partnerships between evidence-based design and architectural morphology.

## Figures and Tables

**Table 1:** Methodology in relation to main research objectives.

Objective (reference to main research objectives)	Methodology	Tools
Personal milieu (I)	Evaluation of patient's needs and compliance to care regime	Semistructured interviews of 30 (for patients) and 23 (for staff) sets of questions
Placemaking for mental health (II)	Data on physical environment and sense of place	Visits, photographical auditing, and architectural blueprints for calculation analogies of areas per use and user group
Domesticity vs institutionalism (I)	Architectural checklist	212 traits on building exterior, layout, and design of interior
Social milieu (I)	Space Syntax analysis	Convex graphs, axial graphs, visibility graphs, and justified graphs

**Table 2:** Institutional features for Wards A and B according to the institutional vs domestic features checklist.

Feature	Ward A		Ward B	
Context and site features	16/22	72.73%	14/22	63.64%
Building features	24/40	60%	24/40	60%
Space and room features	89/150	59.33%	78/150	52%
Total	129/212	60.85%	116/212	54.72%

**Table 3:** Layout similarities and differences for Wards A and B.

	Description	Ward A	Ward B
Similarities	Ground floor	+	+
	Single storey	+	+
	Access to fully protected courtyard	+	+
	Centrally positioned nurse station	+	+
	Centrally positioned clinics	+	+
	Double loaded corridors	(+)	+

Differences	Office area: offices integrated (as opposed to segregation or at the far end)	−	+
	Self-contained ward (vs dependent)	−	+
	Single bedrooms (vs sharing)	−	+
	Toilets: individual (vs shared)	−	+
	Gender segregation: single gendered ward (vs female only area)	−	+

“(+)” signifies that the relevant spatial trait is present but not everywhere. “+” signifies that the specific spatial trait is met and “(−)” that this is not the case.

**Table 4:** Mean institutional percentages for Wards A and B compared to the earlier UK sample.

Facility	Mean
Ward A (2016)	60.85
Ward I (2002)	56
Ward B (2016)	54.72
Ward II (2002)	48
Ward III (2002)	47
Ward IV (2002)	44
Ward V (2002)	26

## Data Availability

The patient's interviews data used to support the findings of this study are restricted by the NRES Committee London ‐ City & East in order to protect patient's privacy. Data are available from Dr Evangelia Chrysikou for researchers who meet the criteria for access to confidential data. The building configuration data used to support the findings of this study are available from the corresponding author upon request.
